# Pressurized Metered-Dose Inhaler Versus Dry Powder Inhaler Adherence Among Individuals with Asthma and COPD

**DOI:** 10.3390/arm93050044

**Published:** 2025-10-11

**Authors:** Dekel Shlomi, Bernice Oberman, Yehonatan Halevy, Shiri Kushnir, Hadas Meir, Yael Reichenberg

**Affiliations:** 1 The Adelson School of Medicine, Ariel University, Ariel 4070000, Israel; 2 Pulmonary Clinic, Dan-Petah-Tiqwa District, Clalit Health Services Community Division, 119 Rothschild St. Petah-Tiqwa, Ramat-Gan 4933355, Israel; 3 Research Unit, Dan-Petah-Tiqwa District, Clalit Health Services Community Division, Ramat-Gan 5120125, Israel; 4 Research Authority, Rabin Medical Center, Beilinson Campus, Petah-Tiqwa 4941492, Israel; 5 Management, Dan-Petah-Tiqwa District, Clalit Health Services Community Division, Ramat-Gan 5120125, Israel

**Keywords:** pressurized metered-dose inhalers: dry-powdered inhalers, adherence, inhaled corticosteroids, long-acting β_2_-agonist, COPD, asthma, asthma–COPD overlap

## Abstract

**Highlights:**

**What are the main findings?**

- Analysis of ICS + LABA inhaler purchases over 12 months showed that overall utilization was very low among individuals with asthma and COPD. The highest purchase rates were observed in those with asthma–COPD overlap.
- Dry powder inhalers (DPIs) were purchased significantly more frequently than pressurized metered-dose inhalers (pMDIs), with once-a-day DPI formulations demonstrating the highest purchase rates.

**What is the implication of the main finding?**

- When prescribing maintenance ICS + LABA inhaler therapy, healthcare professionals should carefully assess actual inhaler purchases at each visit, in addition to evaluating inhaler technique and patient preferences.
- Individuals with high ICS + LABA inhaler purchase rates may either be better-controlled or experiencing more severe symptoms. In such cases, the possibility of asthma–COPD overlap should be considered.

**Abstract:**

*Background:* The core management of most individuals with asthma and COPD is daily treatment with inhalers such as inhaled corticosteroids (ICS) and long-acting bronchodilators. The two main types of inhalers used are pressurized metered-dose inhalers (pMDIs) and dry powder inhalers (DPIs). Different studies have shown low adherence to inhaler treatments among subjects with asthma and COPD. In this study, we explored the differences in adherence between pMDIs and DPIs of combined ICS and long-acting β_2_-agonist inhalers (ICS + LABA) in a large cohort, free from commercial biases. *Methods:* In this historical prospective study, we included all adult subjects with asthma and/or COPD who acquired at least one ICS + LABA inhaler between 2016 and 2019. We carried out propensity score matching and then compared the maximal number of pMDIs and DPIs purchased in any continuous 12 months during the study period. We also compared once-a-day DPIs with twice-a-day DPIs. *Results:* Of the 36,998 matched subjects, 5897 (15.9%) purchased pMDIs. The overall median [IQR] inhalers purchased for pMDIs and DPIs were 1 [1, 4] and 3 [1, 8], respectively; for subjects with asthma, 1 [1, 3] and 2 [1, 6]; for subjects with COPD, 1 [1, 3] and 3 [1, 10]; and for subjects with asthma–COPD overlap, 2 [1, 7] and 6 [2, 12]. For all the comparisons, *p* < 0.001. The once-a-day DPI group had a slight but significantly better adherence than the twice-a-day DPI group. *Conclusions:* For ICS + LABA therapy, the number of DPIs purchased was significantly greater than the number of pMDIs purchased, as well as the once-a-day DPI relative to the other DPIs. Overall, subjects with asthma and/or COPD had low adherence to all inhalers, with the highest adherence observed among subjects with asthma–COPD overlap.

## 1. Introduction

Asthma and chronic obstructive pulmonary disease (COPD) are two prevalent chronic respiratory diseases that significantly affect quality of life and impose a large economic burden [1], and COPD is the fourth leading cause of death worldwide [2]. The core treatment of both asthma and COPD involves the use of inhaled medications. Inhaled corticosteroids (ICS) are the initial maintenance treatment for asthma, with the option of adding long-acting bronchodilators, primarily long-acting β_2_-agonist (LABA) in a single inhaler (ICS + LABA) [3]. On the other hand, long-acting bronchodilators are the initial treatment for COPD, while ICS could be added in cases of exacerbation [4].

The most common types of inhalers are pressurized metered-dose inhalers (pMDIs) and dry powder inhalers (DPIs). Although inhalers are highly effective at reducing asthma and COPD symptoms, exacerbations and death [3,4], many individuals do not use them correctly [5,6,7,8]. A common mistake among pMDI users is a lack of coordination between pressing the inhaler and inhalation, along with shallow or weak inhalation, which reduces drug delivery to the lungs. DPI users also make the mistake of not inhaling forcefully enough to properly release the dose, as well as exhaling into the inhaler before inhaling [9,10]. Misuse of inhalers could lower adherence; thus, to improve treatment outcomes for individuals with COPD and asthma via inhalers, clinicians must be familiar with different inhaler types and their proper usage [8]. A study of subjects with COPD reported that nearly 40% misused their inhaler [11,12], whereas other studies estimated error rates as high as 90% across different devices [13,14]. Among individuals with asthma, approximately 50% do not take their medication as prescribed [3]. In a meta-analysis, Monteiro and colleagues reported that factors such as older age, higher socioeconomic status (SES), greater disease-related literacy, obesity, and better cognitive performance were associated with greater adherence to inhaled therapy, whereas being employed and using multiple inhalers were linked to lower adherence [15].

Several studies have examined the adherence of inhalers according to their type, pMDIs vs. DPIs. Most studies reported better adherence to DPIs [16,17,18]. For example, in a large retrospective study in South Korea, among 13,850 newly diagnosed subjects with asthma initiating ICS + LABA therapy, better adherence was demonstrated for DPIs than for pMDIs, (67% vs. 62%, respectively, *p* < 0.001), as measured by the percentage of days covered (PDC), which is the total number of days the medication was available divided by the length of the observation period [16]. The researchers suggested that, in their opinion, DPIs are easier to use than pMDIs, which could contribute to greater adherence. A study among subjects with asthma treated with ICSs, which used questionnaires, reported a 2.2 times better adherence to DPIs than to pMDIs (95% confidence interval [CI] 1.2–3.8) [18]. Several studies have reported no differences in adherence between pMDIs and DPI [19,20,21]. In contrast, a study by Darbà and colleagues, which used pharmacy data, demonstrated 29% lower adherence to ICS + LABA DPIs than to pMDIs (odds ratio (OR) 0.71, 95% CI 0.521–0.970) [22].

One option to improve adherence is to reduce the number of times a day that the maintenance inhaler must be used. The Global Initiative for Asthma (GINA) recognizes that once instead of twice-a-day inhalers increase adherence (chapter 5) [3]. Studies comparing once-a-day inhalers have shown improved adherence compared with that of twice-a-day inhalers [23,24,25,26,27]. For example, a study by De Keyser and colleagues used electronic medication monitors that captured the time and date of each inhaler actuation and reported that the percentage of subjects who achieved >80% mean daily adherence was greater for once-a-day inhalers than for twice-a-day inhalers in both subjects with asthma (34.3% vs. 23.6%, respectively, *p* < 0.001) and COPD (54.8% vs. 38.6%, *p* < 0.001) [23].

The aim of this study was to explore the differences between the pMDIs and DPIs of subjects with asthma and COPD via actual inhaler purchase data of a large cohort, free from commercial biases. A secondary aim was to compare the adherence between DPIs taken once and twice a day. Our hypothesis is that there is better adherence to DPIs than to pMDIs as well as to once-a-day inhalers than to twice-a-day inhalers.

## 2. Methods

The study was conducted in Clalit Health Services (CHS), Israel’s largest publicly funded Health Maintenance Organization (HMO). In Israel, the medical records of all patients are computerized. We used our electronic medical record databases to explore demographics and diagnoses that were made by the attending physicians as well as pharmaceutical information of inhaler purchases. In this historical prospective (retrospective cohort) study the data of the exposure (the type of inhaler) had been collected at one point in the past and followed (adherence) further but still in the past. The study population consisted of adult male and female subjects with asthma and/or COPD diagnoses who purchased at least one ICS + LABA inhaler between 1 January 2016, and 31 December 2019. The exclusion criterion was subjects under 18 years old. In Israel, there is a universal healthcare system that provides comprehensive medical coverage regardless of SES. Individuals can purchase medication with reduced costs and there are neglectable cost differences between the types of ICS + LABA inhalers.

Subjects with asthma and COPD who require an ICS + LABA inhaler are almost always recommended to use inhalers daily. The use of only as-needed low-dose ICS-formoterol (without maintenance use) was not recommended at the time of the study period. To avoid including subjects who did not purchase ICS + LABA inhalers due to a step down in their treatment or concomitant/intermittent ICS treatment, we excluded subjects who purchased ICS via a single inhaler during the study period. Since a triple inhaler that included a long-acting muscarinic antagonist (LAMA) was not available during the study period, no subjects were treated with other ICS combination therapies. As all inhaler regimens are designed such that only one inhaler is necessary per one month, the expected number of inhalers to be purchased by most subjects for one year is twelve. We could not use the number of prescriptions or compare the differences between prescriptions and purchases since, in our organization, subjects are required to ask their physicians for repeat prescriptions when their medication has run out, a status that is influenced by adherence.

We divided the population into two major groups: subjects who purchased only pMDIs or only DPIs during the study period without changing the type of inhaler. The pMDIs were beclometasone dipropionate + formoterol fumarate (Foster, Cheisi) and fluticasone propionate + formoterol fumarate (Flutiform, Recipharm), and the DPIs were budesonide + formoterol fumarate (Symbicort Turbuhaler, AstraZeneca and DuoResp Spiromax, Teva), fluticasone propionate + salmeterol (Seretide/Advir Diskus, GSK) and fluticasone furoate + vilanterol (Relvar/Breo Ellipta, GSK). For each group, we found the maximum number of pMDIs and DPIs purchased in every continuous 12-month period throughout the entire study (i.e., January 2016 through December 2016, February 2016 through January 2017, March 2016 through February 2017 and so on) and then used the 12-month period with the highest number of inhalers for comparison. We conducted propensity score matching of two groups of subjects, each of whom used only one type of inhaler during the study period, pMDIs or DPIs. The groups underwent exact matching according to age, gender, body mass index (BMI), smoking status, SES and the Charlson comorbidity index (CCI). In addition, we compared the results to those of the crude unmatched groups of the entire study population. For the two methods, we performed subgroup analysis for subjects with just an asthma diagnosis (without coexisting COPD), subjects with just a COPD diagnosis (without coexisting asthma) and subjects with an asthma–COPD overlap diagnosis.

To explore the differences between inhalers used once or twice a day, a separate analysis was conducted in which we compared the once-a-day fluticasone + vilanterol DPI (to all other DPIs. This study was approved by the Institutional Review Board (IRB) of Meir Medical Center, Kfar-Saba, Israel (approval no. 0168-21-COM1, 25 November 2021). Since the study was conducted on the basis of historical data, the IRB approved this study without requesting signed informed consent from the study participants.

For the propensity score analysis, we matched age, gender, BMI group (<18.5, 18.5–<25, 25–<30 and ≥30), smoking background (past or current vs. never), SES (low, medium and high) and CCI. Subjects with missing data were not included in the propensity score analyses. The pMDI and DPI groups were compared using *t* tests or analysis of variance (ANOVA) for continuous variables and chi-square tests for categorical variables. Due to the generally non-normal distributions of the purchase data, we calculated the median ± interquartile ranges (IQRs) of inhaler purchases. We used the Wilcoxon rank sum test to compare between purchases in each group. The statistical analysis was conducted in R—(The R Project for Statistical Computing version 4.2.3 (https://www.r-project.org/ (accessed on 1 October 2025)).

## 3. Results

Among the 66,910 subjects, 6469 (9.7%) purchased pMDIs. The demographic and clinical parameters according to the type of inhaler used in the unmatched study population are presented in Table 1. The mean age was 55 years; 44% were males and 58% were never smokers. The pMDI group had a slightly lower, although not clinically significant, mean age compared to the DPI group (54 vs. 55, respectively, *p* = 0.005), a greater proportion of females (58% vs. 55%, *p* < 0.001) and more subjects with medium SES (69% vs. 63%, *p* < 0.001).

Table 2 presents the demographic and clinical parameters of subjects with asthma, COPD and asthma–COPD overlap according to the type of inhaler in the matched population. Subjects with asthma were younger than subjects with COPD and asthma–COPD overlap (46 vs. 67 and 64, respectively), with higher proportions of females (61% vs. 39% and 57%, respectively), nonsmokers (73% vs. 28% and 50%, respectively), subjects with higher SES (33% vs. 22% and 22%, respectively) and lower mean CCI (3.3 vs. 7.5 and 6.7, respectively). Subjects with asthma also accounted for a lower proportion of subjects with high BMI than subjects with COPD and asthma–COPD overlap (BMI ≥ 30; 26% vs. 31% and 35%, respectively).

**Figure 1:**
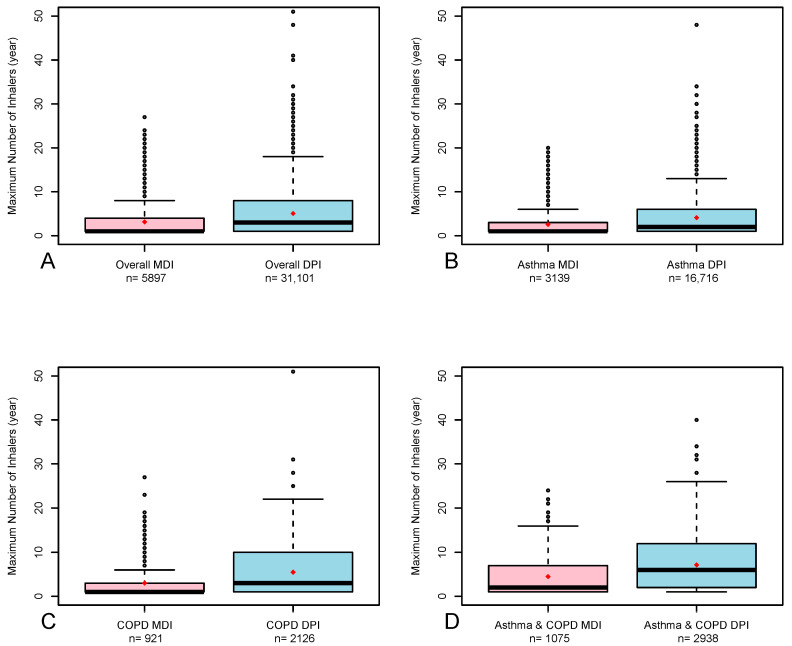
Maximal ICS + LABA pMDI vs. DPI purchases among the entire study population (**A**), subjects with asthma (**B**), subjects with COPD (**C**) and subjects with asthma–COPD overlap (**D**) of the propensity score matching analysis. The boxes represent the distribution from the 25th to the 75th percentiles, the line in it represents the median, the rhombus represents the mean, the whiskers represent the 1.5× interquartile range, and the dots represent the most extreme values. bid, twice-a-day; DPIs, dry powder inhalers; MDIs, metered-dose inhalers; qd, once-a-day.

Subjects with asthma who purchased pMDIs had a slightly greater proportion of females than subjects who purchased DPIs (63% vs. 60.6%, respectively, *p* = 0.005), more subjects with a positive smoking history (28.9% vs. 26.8%, *p* = 0.008), more subjects with a medium SES (65.5% vs. 58.6%, *p* < 0.001) and a slightly greater, although not clinically significant, CCI (3.4 vs. 3.2, *p* = 0.003). Subjects with COPD who purchased pMDIs were slightly younger than subjects who purchased DPIs (66.3 vs. 67.5, respectively, *p* = 0.001), had a significantly greater proportion of females (42% vs. 38.3%, *p* = 0.007), a greater percentage of never-smokers (30.9% vs. 28%, *p* = 0.026), more subjects with medium SES (76.7% vs. 67%, *p* < 0.001) and a slightly lower, but not clinically significant, mean CCI (7.3 vs. 7.6, *p* = 0.024). A greater proportion of subjects with asthma–COPD overlap who purchased pMDIs were females compared to those who purchased DPIs (62% vs. 56%, respectively, *p* < 0.001), and more subjects were in the medium SES (71.6% vs. 68.1%, respectively, *p* = 0.009). Using propensity score methods, 36,998 subjects underwent exact matching, of whom 5897 (15.9%) purchased pMDIs. The medians of inhalers purchased for pMDI and DPI according to the type of analysis (unmatched and propensity score matching) are presented in Table 3. For the exact matched groups, the overall medians [IQR] of inhalers purchased for pMDIs and DPIs were 1 [1, 4] and 3 [1, 8], respectively; for subjects with asthma, the medians [IQR] were 1 [1, 3] and 2 [1, 6]; for subjects with COPD, the medians [IQR] were 1 [1, 3] and 3 [1, 10]; and for subjects with asthma–COPD overlap, the medians [IQR] were 2 [1, 7] and 6 [2, 12]. For all the comparisons, *p* < 0.001. Similar results were found for the unmatched analysis. Figure 1 shows a boxplot of the maximal number of pMDIs and DPIs over 12 months (exact matching) for the entire study population and for subjects with asthma, COPD and asthma–COPD.

**Figure 2:**
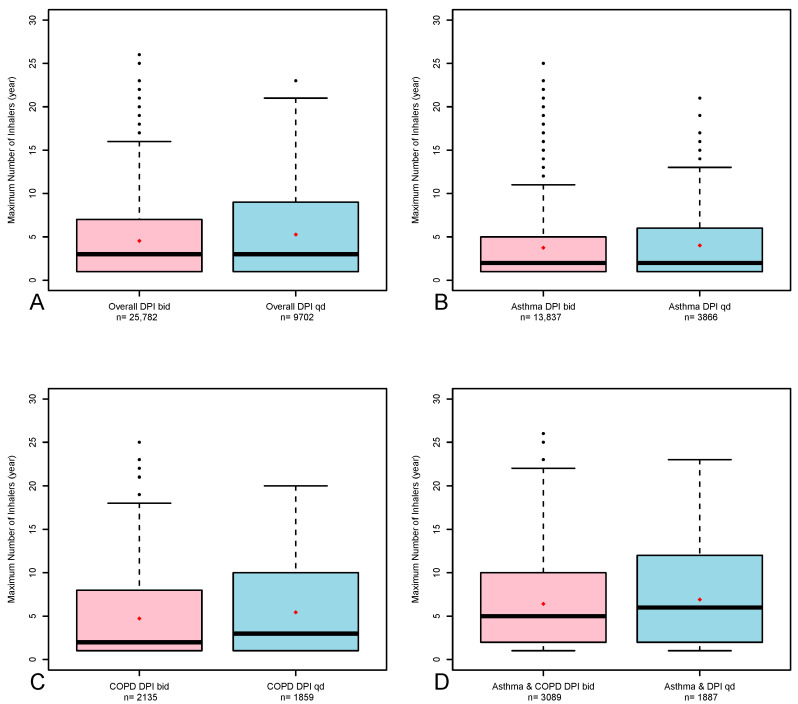
Maximal ICS + LABA once-a-day vs. twice-a-day DPI purchases among the entire study population (**A**), subjects with asthma (**B**), subjects with COPD (**C**) and subjects with asthma–COPD overlap (**D**) of the propensity score matching analysis. The boxes represent the distribution from the 25th to the 75th percentiles, the line in it represents the median, the rhombus represents the mean, the whiskers represent the 1.5× interquartile range, and the dots represent the most extreme values. bid, twice-a-day; DPIs, dry powder inhalers; MDIs, metered-dose inhalers; qd, once-a-day.

Table 4 represents the median inhalers purchased for the once-a-day DPI compared with the twice-a-day DPIs according to the type of analysis (unmatched and propensity score). Of the 50,667 subjects, 35,484 underwent exact matching, of whom 9702 (27.3%) purchased the once-a-day DPI. For the exact-matched groups, the overall medians [IQRs] of inhalers purchased for once and twice-a-day DPIs were 3 [1, 9] and −3 [1, 7], respectively; for subjects with asthma, they were 2 [1, 6] and 2 [1, 5]; for subjects with COPD, they were 3 [1, 10] and 2 [1, 8]; and for subjects with asthma–COPD overlap, they were 6 [2, 12] and 5 [2, 10]. Similar results were found for the unmatched analysis. Figure 2 shows a boxplot of the maximal number of once-a-day DPIs and twice-a-day DPIs over 12 months (exact matching) for the entire study population and for subjects with asthma, COPD and asthma–COPD.

## 4. Discussion

In this study, we explored ICS + LABA inhalers adherence according to the type of inhaler, pMDI and DPI, among subjects with asthma and COPD. We used the maximal purchase for any given 12 months as objective evidence of the maximal possible adherence. For the entire study population, the maximal purchase for any given 12 months during the 4-year study period was low, both for pMDIs (one inhaler for 12 months, 10% of the expected twelve inhalers (see Section 2)), and for DPIs (three inhalers for 12 months, 25% of the expected). Similar results were found for each respiratory disease, asthma and COPD while the adherence of subjects with asthma–COPD overlap was twice that of just asthma or just COPD. DPI purchases were significantly greater than pMDIs for the entire study population as well as for each respiratory disease while the highest adherence (six inhalers in 12 months) was found among subjects with asthma–COPD overlap who purchased DPIs. These differences may be caused by the differences between pMDIs and DPIs usage. For pMDIs to be clinically effective, there is a need for precise coordination between pressing the canister and inhalation, whereas DPIs overcome this need but require greater inspiration force. In addition, the adherence to a once-a-day inhaler was higher than all twice-a-day DPIs for subjects with asthma–COPD overlap (six vs. five, respectively), followed by subjects with COPD (three vs. two, respectively).

The level of adherence in this study (<50%) is considered low in clinical practice and, to the best of our knowledge, compared to several studies [16,26,28]. The results of our study are consistent with those of various review articles, which reported adherence rates of 22–78% among subjects with asthma [8] and 22–93% among subjects with COPD [29]. The large variation in adherence rates among different studies could be related to differences in measurement methods. According to both GINA [3] and GOLD [4], adherence to inhaler therapy is considered low, with rates below 50% of the prescribed medicine.

While several studies reported similar adherence for both types of inhalers (pMDIs and DPIs) [19,20,30,31], our results are consistent with other studies that reported better adherence to DPIs than to pMDIs [16,17,18,26,32]. For example, in a large study by Park and colleagues, among 13,850 newly diagnosed subjects with asthma, better adherence was demonstrated for DPIs (67%) than for pMDIs (62%), as measured by the PDC (*p* < 0.001) [16]. As far as we know, only one study among subjects with COPD reported that the odds of adhering to ICS + LABA DPIs was lower than those of pMDIs (OR = 0.71, 95% CI 0.521–0.970) [22]. Possible explanations could be the smaller sample size (1263 subjects) and older age (mean 70.6 years), for whom pMDIs may be more difficult to use due to coordination issues [22]. Notably, a recent review concluded that when an inhaler is used correctly, there is no considerable difference in adherence between MDIs and DPIs [8].

The better adherence to the once-a-day inhalers in this study is compatible with other studies which demonstrated significantly better adherence with the once-a-day ICS + LABA inhaler than twice-a-day inhalers among subjects with asthma [19,24]. Similar results have been shown among subjects with COPD, in which the adherence to once-a-day inhalers was greater than that of twice-a-day ICS + LABA inhalers [33,34]. In addition, among subjects with COPD, those receiving the once-a-day triple therapy inhaler of ICS + LABA + LAMA, Trelegy (GSK) had better adherence than those receiving the twice-a-day triple therapy inhaler [26,35]. The once-a-day inhalers are the preferred option for treatment of COPD according to the GOLD [4] guidelines especially for individuals with poor adherence or those needing simplicity, and according to the GINA [34] guidelines for individuals with controlled asthma or when maintenance and reliever therapy (MART) is not applicable.

The differences in the adherence between subjects with asthma, COPD and asthma–COPD overlap could be partially explained by the nature of asthma disease, which, in most cases, varies throughout the year; thus, individuals decrease or inappropriately stop inhaler use when the disease is less profound, resulting in lower purchases. Individuals with COPD have more chronic, consistent pulmonary disease, which often progresses and may exacerbate; thus, these individuals need more inhalers. Furthermore, individuals with asthma are usually younger than individuals with COPD (the mean age in this study is 45.5 vs. 67.4, respectively), which may contribute to the lower adherence due to an underestimation of disease severity. On the other hand, among individuals with COPD, adherence may be limited by age-related comorbidities, polypharmacy, or socioeconomic barriers. Asthma–COPD overlaps convey both features, and individuals are usually more symptomatic and experience more exacerbations [3,36]. These individuals may be more motivated by their physicians to use them more consistently along with their higher healthcare utilization, which may improve prescription refills.

To the best of our knowledge, only a few studies have compared adherence among subjects with asthma, COPD, and asthma–COPD overlap, with conflicting results. For example, in a small study by Vanoverschelde and colleagues among 70 subjects, lower adherence was demonstrated among subjects with asthma than among subjects with asthma–COPD overlap (OR 0.13, 95% CI 0.02–0.97) [37]. However, nonsignificant differences were found between subjects with asthma–COPD overlap and subjects with COPD. Other studies have shown different results. For example, a Singaporean study of over 8000 subjects with asthma revealed that those with asthma–COPD overlap were more likely to have poor adherence, measured by the ratio of inhalers dispensed and duration of treatment prescribed (medication possession ratio (MPR) < 50% than subjects with asthma-only (OR 0.75, 95% CI 0.59–0.96)) [19]. A possible explanation for the lower adherence among individuals with asthma–COPD overlap is the need for multiple inhalers with different techniques, which may increase errors and therefore reduce adherence [38].

As expected, there are several limitations to our study. First, this was a historical prospective study; we did not prospectively randomize our study population and could not explore the reasons for which any type of inhaler was prescribed. Furthermore, there were differences in sample size between the small pMDI and the larger DPI groups. Nevertheless, we tried to overcome these issues by using propensity score matching according to the main demographic and clinical parameters. Another limitation is that information about inhaler adherence is derived from purchasing data, which differs from the actual use of the drug. However, since the study explored the maximal purchase during any 12 continuous months for several years, the purchase represents the maximal possibility for using the inhalers. Given the low overall adherence and cost-sharing, purchases were probably close to actual usage. In addition, we did not analyze different dosages of the ICS + LABA inhalers since this approach is complex and variable. We believe that the dosage should be changed according to symptoms, particularly for individuals with asthma, and by changing the dose, the factor of efficacy as a contributor to adherence is reduced. Moreover, differences in adherence between MDIs and DPIs are likely influenced by several confounding factors beyond device type. These include patient characteristics (such as age, socioeconomic and educational status, cognitive function and dexterity), disease-related aspects (asthma vs. COPD and disease severity), treatment complexity, individual preferences for inhaler type and technique-related issues (such as coordination with pMDIs, the use of spacers, or the inspiratory force required for DPIs). Thus, observed discordance in adherence should be interpreted within this broader context. Nevertheless, this study measured the net outcome of these factors. Medical personnel should be aware of low adherence and refer to the wide aspects of this complex issue on a personal level. These limitations should be considered with respect to the strengths of the study. First, this is an independent study that is not related to any pharmaceutical company and was conducted using real-life data from a large population of approximately 67,000 people. The study also compared different respiratory diseases and, in particular, explored subjects with asthma–COPD overlap, for whom data are lacking. Finally, we could effectively adjust this large population according to age, sex, BMI, smoking status, SES and the CCI. Furthermore, the consistent results and their correspondence with those of most of the previous literature strengthen our findings.

For ICS + LABA therapy, maximal DPI adherence, measured by maximal purchases during any 12 months over the study period, was significantly greater than that of pMDIs among subjects with asthma, COPD and asthma–COPD. The once-a-day DPI demonstrated better adherence than the twice-a-day DPIs. Overall, subjects with either asthma or COPD have low adherence to all inhalers, with the highest adherence reported was among subjects with asthma–COPD overlap which may suggest greater disease symptoms. Physicians, nurses and pharmacists should thoroughly examine the actual purchase of inhalers along with the technical usage and personal preferences of their patients. Another consideration is the environmental cost of inhalers. pMDIs have a significantly greater carbon footprint than DPIs, which do not use propellant gases that contribute to greenhouse emissions. On the other hand, DPIs production and distribution involve fossil fuel-based plastics, energy-intensive drug formulations, packaging waste, and end-of-life disposal challenges [39].

## Figures and Tables

**Table 1:** Characteristics of the study population according to MDIs and DPIs purchased.

	Total	MDIs	DPIs	*p*
**No**	66,910	6469	60,441	
**Age**				
Mean (SD)	54.9 (19.6)	54.3 (19.5)	55.0 (19.6)	0.005
**Gender (%)**				
Male	29,687 (44.4)	2696 (41.7)	26,991 (44.7)	<0.001
**BMI (%)**				
<18.5	1706 (2.5)	161 (2.5)	1545 (2.6)	0.403
18.5–<25	22,687 (33.9)	2180 (33.7)	20,507 (33.9)	
25–<30	22,860 (34.2)	2170 (33.5)	20,690 (34.2)	
≥30	19,657 (29.4)	1958 (30.3)	17,699 (29.3)	
**Smoking status (%)**				
never	38,849 (58.1)	3754 (58.0)	35,095 (58.1)	0.968
past or current	28,061 (41.9)	2715 (42.0)	25,346 (41.9)	
**Socioeconomic status (%)**				
Low	5797 (8.7)	433 (6.7)	5364 (8.9)	<0.001
Medium	42,493 (63.5)	4483 (69.3)	38,010 (62.9)	
High	18,620 (27.8)	1553 (24.0)	17,067 (28.2)	
**Charlson comorbidity index (SD)**	5 (3.8)	5 (3.8)	5 (3.8)	0.147

COPD, chronic obstructive pulmonary disease; DPIs, dry powder inhalers; MDIs, metered-dose inhalers; SD, standard deviation; IQR, interquartile range; BMI, body mass index.

**Table 2:** Characteristics of the asthma, COPD and asthma–COPD overlap subjects according to MDIs and DPIs purchased.

	Asthma				COPD				Asthma–COPD Overlap			
	Total	pMDIs	DPIs	*p*	Total	pMDIs	DPIs	*p*	Total	pMDIs	DPIs	*p*
**No (%)**	35,431	3601	31,830		12,456	1408	11,048		19,023	1460	17,563	
**Age**												
Mean (SD)	45.5 (18.4)	45.8 (18.5)	45.4 (18.4)	0.311	67.4 (12.3)	66.3 (12.5)	67.5 (12.3)	0.001	64.4 (16.0)	63.7 (16.5)	64.5 (16.0)	0.074
**Gender (%)**												
Male	13,872 (39.2)	1331 (37.0)	12,541 (39.4)	0.005	7631 (61.3)	816 (58.0)	6815 (61.7)	0.007	8184 (43.0)	549 (37.6)	7635 (43.5)	<0.001
**BMI (%)**												
<18.5	981 (2.8)	96 (2.7)	885 (2.8)	0.623	361 (2.9)	42 (3.0)	319 (2.9)	0.149	364 (1.9)	23 (1.6)	341 (1.9)	0.714
18.5–<25	13,722 (38.7)	1379 (38.3)	12,343 (38.8)		3792 (30.4)	408 (29.0)	3384 (30.6)		5173 (27.2)	393 (26.9)	4780 (27.2)	
25–<30	11,591 (32.7)	1165 (32.4)	10,426 (32.8)		4444 (35.7)	485 (34.4)	3959 (35.8)		6825 (35.9)	520 (35.6)	6305 (35.9)	
≥30	9137 (25.8)	961 (26.7)	8176 (25.7)		3859 (31.0)	473 (33.6)	3386 (30.6)		6661 (35.0)	524 (35.9)	6137 (34.9)	
**Smoking status (%)**												
never	25,857 (73.0)	2560 (71.1)	23,297 (73.2)	0.008	3531 (28.3)	435 (30.9)	3096 (28.0)	0.026	9461 (49.7)	759 (52.0)	8702 (49.5)	0.078
past or current	9574 (27.0)	1041 (28.9)	8533 (26.8)		8925 (71.7)	973 (69.1)	7952 (72.0)		9562 (50.3)	701 (48.0)	8861 (50.5)	
**Socioeconomic status (%)**												
Low	2697 (7.6)	218 (6.1)	2479 (7.8)	<0.001	1206 (9.7)	96 (6.8)	1110 (10.0)	<0.001	1894 (10.0)	119 (8.2)	1775 (10.1)	0.009
Medium	21,011 (59.3)	2357 (65.5)	18,654 (58.6)		8484 (68.1)	1080 (76.7)	7404 (67.0)		12,998 (68.3)	1046 (71.6)	11,952 (68.1)	
High	11,723 (33.1)	1026 (28.5)	10,697 (33.6)		2766 (22.2)	232 (16.5)	2534 (22.9)		4131 (21.7)	295 (20.2)	3836 (21.8)	
**Charlson comorbidity index (SD)**	3.3 (2.9)	3.4 (3)	3.2 (2.9)	0.003	7.5 (3.5)	7.3 (3.6)	7.6 (3.5)	0.024	6.7 (3.7)	6.6 (3.7)	6.7 (3.7)	0.2

COPD, chronic obstructive pulmonary disease; DPIs, dry powder inhalers; MDIs, metered-dose inhalers; SD, standard deviation; IQR, interquartile range; BMI, body mass index.

**Table 3:** Comparisons between MDI and DPI purchases of the entire population and according to the respiratory disease using unmatched and propensity score analyses.

	No Matching			Propensity Score Exact Matching		
	n	Median [IQR]	*p* Value	n	Median [IQR]	*p* Value
**Entire population**						
	**66,910**			**36,998**		
**MDIs (%)**	6469 (9.7%)	1 [1, 4]	<0.001	5897 (15.9)	1 [1, 4]	<0.001
**DPIs (%)**	60,441 (90.3)	3 [1, 9]		31,101 (84.1)	3 [1, 8]	
**Only asthma**						
	**35,431**			**19,855**		
**MDIs (%)**	3601 (10.2)	1 [1, 3]	<0.001	3139 (15.8)	1 [1, 3]	<0.001
**DPIs (%)**	31,830 (89.8)	2 [1, 7]		16,716 (84.2)	2 [1, 6]	
**Only COPD**						
	**12,456**			**3047**		
**MDIs (%)**	1408 (11.3)	1 [1, 3]	<0.001	921 (30.2)	1 [1, 3]	<0.001
**DPIs (%)**	11,048 (88.7)	3 [1, 10]		2126 (69.8)	3 [1, 10]	
**Asthma–COPD overlap**						
	**19,023**			**4013**		
**MDIs (%)**	1460 (7.7)	2 [1, 7]	<0.001	1075 (26.8)	2 [1, 7]	<0.001
**DPIs (%)**	17,563 (92.3)	7 [2, 12]		2938 (73.2)	6 [2, 12]	

COPD, chronic obstructive pulmonary disease; DPIs, dry powder inhalers; IQR, interquartile range; MDIs, metered-dose inhalers.

**Table 4:** Comparisons between once and twice-a-day DPI purchases of the entire population and according to the respiratory disease using unmatched and propensity score analyses.

	No Matching			Propensity Score Exact Matching		
	n	Median [IQR]	*p* Value	n	Median [IQR]	*p* Value
**Entire population**						
	**50,667**			**35,484**		
**Once-a-day (%)**	11,746 (23.2)	3 [1, 10]		9702 (27.3)	3 [1, 9]	
			<0.001			<0.001
**Twice-a-day (%)**	38,921 (76.8)	3 [1, 7]		25,782 (72.7)	3 [1, 7]	
**Only asthma**						
	**27,704**			**17,703**		
**Once-a-day (%)**	4751 (17.1)	2 [1, 6]		3866 (21.8)	2 [1, 6]	
			<0.001			0.053
**Twice-a-day (%)**	22,953 (82.9)	2 [1, 6]		13,837 (78.2)	2 [1, 5]	
**Only COPD**						
	**9438**			**3994**		
**Once-a-day (%)**	3762 (39.9)	3 [1, 10]		1859 (46.8)	3 [1, 10]	
			<0.001			0.001
**Twice-a-day (%)**	5676 (60.1)	2 [1, 8]		2135 (53.2)	2 [1, 8]	
**Asthma–COPD overlap**						
	**13,525**			**4976**		
**Once-a-day (%)**	3233 (23.9)	6 [2, 12]		1887 (38.7)	6 [2, 12]	
			<0.001			0.01
**Twice-a-day (%)**	10,292 (76.1)	5 [2, 10]		3089 (63.3)	5 [2, 10]	

COPD, chronic obstructive pulmonary disease; DPIs, dry powder inhalers; IQR, interquartile range; MDIs, metered-dose inhalers.

## Data Availability

Except for statistical analysis, the data underlying this article cannot be shared publicly due to privacy issues such as personal details of the study participants. The data will be shared at reasonable requests to the corresponding author.

## Abbreviations

BMI	body mass index
CHS	Clalit Health Services
CCI	Charlson comorbidity index
CI	confidence interval
COPD	chronic obstructive pulmonary disease
DPI	dry powder inhaler
GINA	Global Initiative for Asthma
GOLD	Global Initiative for Chronic Obstructive Lung Diseases
HMO	health maintenance organization
ICS	inhaled corticosteroids
IRB	institutional review board
IQR	interquartile range
LABA	long-acting β2-agonist
LAMA	long-acting muscarinic antagonist
MPR	medication possession ratio
OR	odds ratio
PDC	proportion of days covered
pMDI	pressurized metered-dose inhaler
SD	standard deviation
SES	socioeconomic status
